# From Pixels to Precision: A Survey of Monocular Visual Odometry in Digital Twin Applications †

**DOI:** 10.3390/s24041274

**Published:** 2024-02-17

**Authors:** Arman Neyestani, Francesco Picariello, Imran Ahmed, Pasquale Daponte, Luca De Vito

**Affiliations:** Department of Engineering, University of Sannio, 82100 Benevento, Italy; neyestani@unisannio.it (A.N.); fpicariello@unisannio.it (F.P.); iahmed@unisannio.it (I.A.); daponte@unisannio.it (P.D.)

**Keywords:** monocular, localization, feature based, odometry, survey, machine learning, deep learning, measurement

## Abstract

This survey provides a comprehensive overview of traditional techniques and deep learning-based methodologies for monocular visual odometry (VO), with a focus on displacement measurement applications. This paper outlines the fundamental concepts and general procedures for VO implementation, including feature detection, tracking, motion estimation, triangulation, and trajectory estimation. This paper also explores the research challenges inherent in VO implementation, including scale estimation and ground plane considerations. The scientific literature is rife with diverse methodologies aiming to overcome these challenges, particularly focusing on the problem of accurate scale estimation. This issue has been typically addressed through the reliance on knowledge regarding the height of the camera from the ground plane and the evaluation of feature movements on that plane. Alternatively, some approaches have utilized additional tools, such as LiDAR or depth sensors. This survey of approaches concludes with a discussion of future research challenges and opportunities in the field of monocular visual odometry.

## 1. Introduction

Exploring unknown environments is a complex challenge that has engaged researchers across various fields. The intricacies of navigating in uncharted territories require the integration of multiple approaches and the development of sophisticated methodologies. Among these, the measurement of accurate camera movements to update the digital twin model of structures plays a significant role, which will be briefly explained in Section 1.1. Modern navigation systems are often multi-modal, merging information collected from various methods to achieve enhanced precision. Within this complex interplay, Visual Simultaneous Localization and Mapping (VSLAM) has emerged as a vital tool in computer vision, robotics, and augmented reality.

VSLAM represents an innovative approach to navigation, addressing the inherent drift problem through the intelligent combination of camera information with an environment map. This map, updated incrementally as an agent such as a robot that moves through the environment, facilitates the accurate and real-time estimation of the surroundings. The significance of this technology is further underscored by its reliance on the accuracy of geometrical measurements, which are pivotal to the localization system. This mechanism aids in the consistent update of models, often evaluated through the periodic acquisitions of camera images, identification of model elements, and assessment of changes over time.

The intricate design of modern navigation systems is underscored by their reliance on the integration of various methods, a process akin to data fusion. This integration involves merging information from different sources to achieve greater accuracy. The role of Visual Simultaneous Localization and Mapping (VSLAM) is particularly significant in this framework. Employed in fields like computer vision, robotics, and augmented reality, VSLAM goes beyond merely combining camera visuals with environmental layouts. Its true value emerges in the continuous refinement and updating of data, enabling robots or agents to adeptly navigate through the ever-changing and unpredictable terrains of unfamiliar settings.

VSLAM’s capabilities are broadened through the use of one or more video cameras to reconstruct a 3D map of an often unknowable environment [1] and to gauge the egomotion—defined as the 3D shifting within space—of the camera itself [2]. The video cameras used in VSLAM systems are essential for applications, like markerless augmented reality and autonomous robotic navigation. When compared with general SLAM that uses sensors like Light Detection and Ranging (LiDAR), VSLAM’s reliance on video cameras brings added advantages [3]. Video cameras are often smaller, less expensive, and carry rich visual information, making them suitable for platforms with limited payloads and lower costs than LiDar or an RGB-D camera [4,5].

Visual odometry (VO) and VSLAM are two closely related techniques that are used to determine a robot or machine’s location and orientation through the analysis of corresponding camera images. Both techniques can utilize a monocular camera, but they have distinct characteristics and objectives [6,7,8].

VO is a technique primarily focused on the real-time tracking of a camera’s trajectory, offering local or relative estimates of the position and orientation. This process is a part of a broader category known as relative visual localization (RVL). RVL encompasses methods like VO, which estimate the motion of robots (both rotation and translation) by localizing themselves within an environment. This localization is achieved by analyzing the differences between sequential frames captured by the camera. One of the key techniques used in VO is Windowed optimization. Windowed optimization is a process that refines the local estimation of the camera trajectory by considering a certain number of previous frames or ‘window’ of frames. This approach helps to improve the accuracy of pose predictions derived from the analysis of image sequences [9,10].

On the other hand, VSLAM delivers a global and consistent estimate of the path of a device, a process often referred to as absolute visual localization (AVL). AVL provides a pose of a vehicle that is often represented by a six-degrees-of-freedom (DoFs) pose vector (x,y,z,φ,θ,ψ) [6]. VSLAM has the ability to reduce drift through techniques, like adjusting the bundle and detecting loop closure [11]. The key difference is that VO is about relative positioning without an understanding of the larger environment, while VSLAM involves both mapping the environment and locating the device within that map.

Loop closure is a sub-algorithm of SLAM that identifies previously visited locations and uses them to correct the accumulated errors in the robot’s pose estimation [12]. The main goal in loop closure is to detect when the robot is observing a previously explored scene so that additional constraints can be added to the map [13]. This is crucial in ensuring the consistency of the map and the accuracy of the robot’s location. The similarities between VO and VSLAM persist until a loop is closed, after which their functions diverge [2,14,15].

Furthermore, VSLAM’s capacity to continuously update the initial map of the environment based on sensor measurements contributes to its adaptability, enabling it to reflect changes, such as new objects or variations in lighting conditions. This makes VSLAM a more comprehensive solution for mapping and localization tasks in dynamic environments. Monocular VO represents an essential component in the field of robotic navigation and computer vision, enabling the real-time estimation of a camera’s trajectory within an environment. Through the meticulous tracking of visual features in consecutive camera frames, VO generates insights into the camera’s motion, a task that has both theoretical and practical significance [15,16,17].

From a practical standpoint, VO has a wide range of applications. It is used in mobile robots, self-driving cars, unmanned aerial vehicles, and other autonomous systems to provide robust navigation and obstacle avoidance capabilities [18,19].

### 1.1. Visual Odometry for Digital Twin

**Figure 1:**
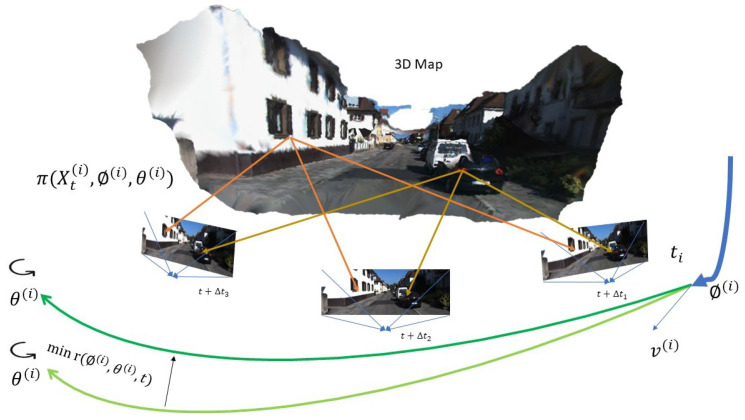
The diagram depicts the camera maneuvers used to update the digital twin representation. The starting position $v(t_{i})$ of the present path segment is depicted by a blue arrow at the moment $t_{i}$. The position for each snapshot is calculated using the parameters $\theta_{t}$. Subsequently, the map points are reprojected onto every snapshot, and the reprojection discrepancy $r(\Phi \left(i\right),\theta_{t},t)$ is reduced to ascertain the accurate path [25].

The accurate measurement of camera movements is crucial for updating the digital twin model of structures. This process involves the use of VO and other techniques to capture and analyze camera images, which are then used to update the digital twin model (Figure 1). One method employed to achieve this involves multi-camera systems. Research examining Blender’s application in designing camera-based measurement systems revealed that it allows for the flexible and rapid modeling of camera positions for motion tracking, which helps determine their optimal placements. This approach significantly cuts down setup times in practical scenarios. The methodologies focus on building an entire virtual camera, encompassing everything from the original camera sensor to the radiometric characteristic of an actual camera [20]. The study focuses on developing virtual representations of multi-camera measurement systems using Blender. It investigates whether these virtual cameras in Blender can perceive and measure objects as effectively as real cameras in similar conditions. Blender, an open-source software for three-dimensional animation, also serves as a simulation tool in metrology. It allows for the creation of numerical models instrumental in the design and enhancement of camera-based measurement systems.

In a separate study, the Digital Twin Tracking Dataset (DTTD) was introduced for Extended-Range Object Tracking. This dataset, comprising scenes captured by a single RGB-D camera tracked by a motion capture system, is tailored to pose estimation challenges in digital twin applications [21].

Regarding geometric change detection in digital twins, an object’s pose is estimated from its image and 3D shape data. This technique is crucial for pose estimation [22]. Likewise, for the digital twin modeling of composite structures, the Azure Kinect camera is utilized to capture both depth and texture information [23]. Drone inspection imagery is instrumental in forming operational digital twins for large structures, enabling the creation and updating of digital twin models based on high-quality drone-captured images [24]. In summary, the precise measurement of camera movements is key in updating digital twin models of structures. Techniques like monocular VO, multi-camera measurement, and drone imagery contribute significantly to producing detailed and accurate digital twin models.

This paper aims to provide a comprehensive examination of the state of the art in various approaches to VO, with an emphasis on recent developments in the use of monocular cameras. By presenting a thorough analysis, it contributes to a broader understanding of this complex and rapidly evolving field. The remaining sections are structured as follows: Section 2 outlines the fundamental concepts and general procedure for VO implementation, while Section 3 explores the research challenges inherent in VO implementation. Section 6 discusses the positioning uncertainty assessment provided by monocular visual odometry. The subsequent sections offer overviews of traditional methods and machine learning-based approaches, culminating in a discussion of future research challenges and opportunities. The articulation of these elements provides a solid foundation for scholars and practitioners interested in navigating the rich and multifaceted landscape of VO and VSLAM technologies.

## 2. Basics of Monocular Visual Odometry

From a theoretical perspective, VO is a complex problem that involves the intersection of multiple disciplines, including computer vision, robotics, and mathematics. It requires the development and application of algorithms that can accurately track visual features and estimate camera motion from a sequence of images [18]. This involves dealing with challenges such as scale ambiguity in monocular systems, where the trajectory of a monocular camera can only be recovered up to an unknown scale factor [19]. Theoretical advancements in VO can contribute to a deeper understanding of these challenges and the development of more effective solutions.

The foundational algorithm of VO, commencing after compensating for camera distortion based on parameters estimated during a calibration phase, can be conceptually divided into several sequential steps, each of which contributes to the overarching objective of motion and trajectory estimation:

1. *Feature detection*: In the initial phase of VO, the focus is on identifying and capturing key visual features from the first camera frame, which are essential for tracking movements across frames. This process, fundamental for the accurate monitoring of camera movement, traditionally relies on algorithms like Harris, SIFT, ORB, and BRISK to pinpoint precise and durable features, such as corners or edges. However, it is crucial to expand beyond these to include line and planar features, which have proven to be invaluable in enhancing the robustness and completeness of feature detection and matching in monocular VO systems. These additions are essential for capturing the full complexity and variety of real-world environments [26,27,28,29].
2. *Feature tracking*: Following feature detection, the VO algorithm focuses on tracking these identified features across consecutive frames. This tracking establishes correspondences between features in successive frames, creating a continuity that facilitates motion analysis. Techniques such as KLT (Kanade–Lucas–Tomasi) tracking or optical flow have proven effective in this context, enabling accurate alignment and correspondence mapping [30].
3. *Motion estimation*: With the correspondences between features in consecutive frames established, the next task is to estimate the camera’s motion. This process involves mathematical techniques, such as determining the essential matrix or, if needed, the fundamental matrix. These methods leverage the correspondences to ascertain the relative motion between frames, providing a snapshot of how the camera’s position changes over time [31].
4. *Triangulation*: Based on the estimated camera motion, the algorithm then moves to determine the 3D positions of the tracked features by triangulation. This technique involves estimating the spatial location of a point by measuring angles from two or more distinct viewpoints. The result is a three-dimensional mapping of features that adds depth and context to the analysis [32].
5. *Trajectory estimation*: The final step in the basic VO algorithm involves synthesizing the previously gathered information to estimate the camera’s overall trajectory within the environment and map the surroundings. This composite task draws upon both the estimated camera motion from step (iii) and the 3D positioning of the tracked features from step (iv). Together, these elements coalesce into a coherent picture of the camera’s path, contributing to a broader understanding of the spatial context [33].

In summary, the basic algorithm for VO is a multi-step process that artfully combines detection, tracking, estimation trajectory, and triangulation to provide a nuanced understanding of camera motion within an unknown environment. By progressing through these distinct yet interrelated phases, VO offers a versatile and valuable tool in the quest to navigate and interpret complex spatial environments. Its contributions extend across various domains, and its underlying methodologies continue to stimulate research and innovation in both theoretical and applied contexts.

## 3. Research Challenges in Monocular Visual Odometry

Monocular visual odometry (VO) represents a sophisticated domain characterized by exceptional achievements and compelling intricacy. The sources of uncertainty can significantly affect the accuracy and reliability of positioning and navigation solutions provided by VO systems. The advancements achieved in this field have substantially contributed to the evolution of robotics, augmented reality, and navigation systems, yet substantial challenges persist. These obstacles highlight the complex constitution of VO and propel ongoing scholarly inquiry and innovation in the discipline.

- **Feature Detection and Tracking**: The efficacy of monocular VO hinges on the precise detection and tracking of image features, which are critical measurements in the VO process. Uncertainties in these measurements arise under conditions of low-texture or nondescript environments, which can be exacerbated by inadequate lighting and complex motion dynamics, challenging the robustness of feature-matching algorithms and leading to measurement inaccuracies [34].
- **Motion Estimation**: Robust motion estimation is central to VO, with its accuracy contingent upon the reliability of feature correspondence measurements. Uncertainty in these measurements can occur due to outliers from incorrect feature matching and drift resulting from cumulative errors in successive estimations, significantly complicating the attainment of precise motion measurements [35].
- **Non-static Scenes**: The premise of VO algorithms typically involves the assumption of static scenes, thereby simplifying the measurement process. However, uncertainty is introduced in dynamic environments where moving objects induce variances in the measurements, necessitating advanced methods to discern and correctly interpret camera motion amidst these uncertainties.
- **Camera Calibration**: The accurate calibration of camera parameters is foundational for obtaining precise VO measurements. Uncertainties in calibration—due to factors such as environmental temperature changes, light conditions, lens distortions, or mechanical misalignments—can significantly distort measurement accuracy, impacting the reliability of subsequent VO estimations [36].
- **Scaling Challenges**: In VO, the lack of an absolute reference frame introduces uncertainty in scale measurements, a pivotal component for establishing the camera’s absolute trajectory. Inaccuracies in these scale measurements can arise from ambiguous geometries, limited visual cues, and the monocular nature of the data, which may lead to scale drift and wrong trajectory computations [37].
- **Ground Plane Considerations**: The ground plane is often used as a reference in VO measurements for scale estimation. However, uncertainties in these measurements can be attributed to ambiguous ground features, variable lighting conditions that affect feature visibility, and scaling complexities relative to object heights, challenging the accuracy of VO scale measurements [38].
- **Perspective Projection**: The perspective projection in monocular VO introduces inherent uncertainties due to the transformation of 3D scenes into 2D images, leading to challenges such as depth information loss and scale ambiguity. This projection results in the foreshortening and distortion of objects, complicating the estimation of relative distances and sizes. Additionally, the overlapping of features in the 2D plane can cause occlusions, disrupting the feature tracking crucial for motion estimation. The projection of 3D points onto a 2D plane also introduces feature perspective errors, especially when features are distant from the camera center or when the camera is close to the scene.
- **Timestamp Synchronization Uncertainty**: This type of uncertainty arises when there are discrepancies in the timing of the data capture and processing among different components of a system, such as cameras, inertial measurement units (IMUs), and LiDAR scanners. In systems that rely on precise timing for data integration and analysis, such as visual–inertial navigation systems, this uncertainty can significantly impact accuracy [9].

In summary, the field of monocular VO offers a rich landscape of technological possibilities, bounded by multifaceted challenges that span detection, estimation, scaling, real-time processing, and more. In another aspect, noise sensitivity refers to the impact of image noise on the performance of VO algorithms, which can degrade the accuracy of feature extraction and matching, ultimately affecting the estimated camera trajectory [39]. An uncertainty assessment is essential for evaluating the reliability of the estimated camera trajectory in VO. While traditional VO approaches often provide an analytical formula for uncertainty, this remains an open challenge for machine learning-based VO methods [40].

Data synchronization is another important aspect in monocular VO, especially when integrating data from multiple sensors, such as cameras and inertial measurement units (IMUs) [41]. Proper synchronization ensures that the data from different sensors are accurately aligned in time, allowing for more precise and reliable trajectory estimation. In some cases, hardware synchronization is used to align the data from different sensors to a common clock, ensuring accurate data fusion and improved VO performance [41].

Achieving real-time performance is imperative for VO applications, yet it poses a challenge due to the computational intensity required for processing measurements. Uncertainty in real-time performance metrics can stem from variable environmental conditions that impact the speed and accuracy of feature detection and matching computations. For example, imagine a self-driving car using VO for navigation. Achieving real-time performance is crucial because the car needs to make immediate decisions based on its surroundings. However, this is challenging due to the heavy computational load required to process the camera’s measurements quickly and accurately.

These challenges not only define the current state of VO but also delineate the paths for future research and exploration. By grappling with these complexities, the scientific community continues to pave the way for more nuanced and powerful applications of VO, extending its reach and impact across various domains. A summary of the various approaches and their implications can be found in Table 1, offering a succinct overview of the literature’s breadth and depth.

## 4. Traditional Approaches

The scientific literature is rife with diverse methodologies aiming to overcome the challenges outlined in the preceding section, particularly focusing on the problem of accurate scale estimation. This issue has typically been addressed through the reliance on knowledge regarding the height of the camera from the ground plane and the evaluation of feature movements on that plane. Alternatively, some approaches have utilized additional tools, such as LiDAR or depth sensors.

Within the domain of autonomous driving, precise vehicle motion estimation is a crucial concern. Various powerful algorithms have been devised to address this need, although most commonly, they depend on binocular imagery or LiDAR measurements. In the following paragraphs, an overview of some prominent works associated with the scaling challenge is provided, highlighting different strategies and technologies.

Tian et al. [55] made a significant contribution by developing a lightweight scale recovery framework for VO. This framework hinged on a ground plane estimate that excelled in both accuracy and robustness. By employing a meticulous ground point extraction technique, the framework ensured precision in the ground plane estimate. Subsequently, these carefully selected points were aggregated through a local sliding window and an innovative ground point aggregation algorithm. To translate the aggregated data into the correct scale, a Random Sample Consensus (RANSAC)-based optimizer was employed. This optimizer solved a least-squares problem, fine-tuning parameters to derive the correct scale, and thus displaying the marriage of optimization techniques and spatial analysis. The parameters for this fine-tuning are likely chosen based on experimental results to achieve the best performance

H. Lee et al. [43] presented a VO system using a downward-facing camera. This system, designed for mobile robots, integrates feature extraction, a novel velocity-aware masking algorithm, and a nonconvex optimization problem to enhance pose estimation accuracy. It employs cost-effective components, including an LED for illumination and a ToF sensor, to improve feature tracking on various surfaces. The methodology combines efficient feature selection with global optimization for motion estimation, demonstrating improved accuracy and computational efficiency over the existing methods. The authors claimed the experimental results validated its performance in diverse environments, showcasing its potential for robust mobile robot navigation.

B. Fang et al. [46] proposed a method for enhancing monocular visual odometry through the integration of LiDAR depth information, aiming to overcome inaccuracies in feature-depth associations. The methodology involves a two-stage process: initial pose estimation through photometric error minimization and pose refinement using point-line features with photometric error minimization for more accurate estimation. It employs ground and plane point segmentation from LiDAR data, optimizing frame-to-frame matching based on these features, and incorporating multi-frame optimization to reduce drift and enhance accuracy. Based on the authors’ claim, the approach demonstrates improved pose estimation accuracy and robustness across diverse datasets, indicating its effectiveness in real-world scenarios.

Chiodini et al. [42] expanded the improvement on scale estimation by demonstrating a flexible sensor fusion strategy. By merging data from a variety of depth sensors, including Time-of-Flight (ToF) cameras and 2D and 3D LiDARs, the authors crafted a method that broke free from the constraints of sensor-specific algorithms that pervade much of the literature. This universal applicability is particularly significant for mobile systems without specific sensors. The proposed approach optimized camera poses and landmark maps using depth information, clearing up the scale ambiguity and drift that can be encountered in monocular perception.

LiDAR–monocular visual odometry (LiMo) was presented by Graeter et al. [14]. This novel algorithm capitalizes on the integration of data from a monocular camera and LiDAR sensor to gauge vehicle motion. By leveraging LiDAR data to estimate the motion scale and provide additional depth information, LiMo enhances both the accuracy and robustness of VO. Real-world datasets were utilized to evaluate the proposed algorithm, and it exhibited marked improvements over other state-of-the-art methods. The potential applications of LiMo in fields like autonomous driving and robotics underscore the relevance and impact of this research.

To mitigate the influence of outliers on feature detection and matching and enhance motion estimation, other researchers introduced data fusion with inertial measurements. This visual–inertial odometry (VIO) integrated system is exemplified in works like Shan et al. [44], which brought together LiDAR, visual, and inertial measurements in a tightly coupled LiDAR–visual–inertial (LVI) odometry system. This holistic fusion, achieved through a novel smoothing and mapping algorithm, elevates the system’s accuracy and robustness. The proposal also introduced an innovative technique for estimating extrinsic calibration parameters, further optimizing performance for applications like autonomous driving and robotics.

Wisth et al. [45] and ORB-SLAM3 [47] further illustrated the technological advances in multi-sensor odometry systems and real-time operation in various environments. The use of factor graphs, dense mapping systems, and various sensors such as IMUs, visual sensors, and LiDAR highlights the multifaceted approaches to challenges in motion and depth estimation.

**Figure 2:**
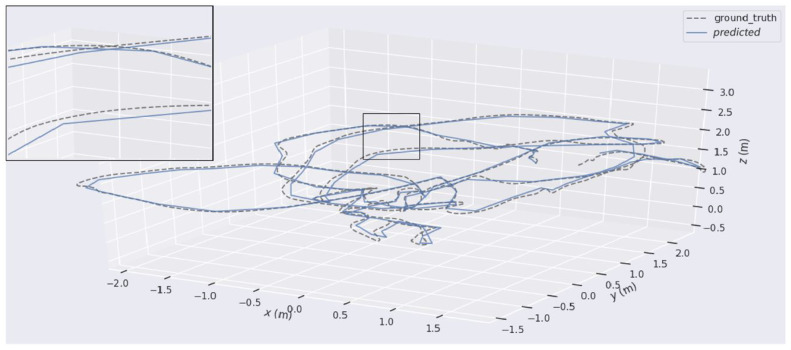
This figure demonstrates the accuracy and effectiveness of the proposed nonlinear optimization-based monocular dense mapping system of VIO [56].

**Figure 3:**
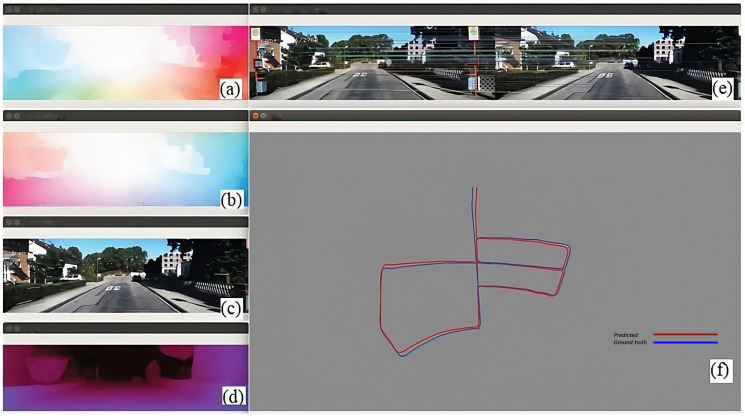
Images in the left column, arranged vertically, are as follows: (**a**) optical flow map in forward order, (**b**) optical flow map in reverse order, (**c**) points of instantaneous optical flow superimposed on the original image, (**d**) map showing monocular depth, (**e**) map illustrating the matching of key points in a pair of images, and (**f**) map depicting the reconstructed trajectory, where the estimated path is indicated by a blue line [51].

Chuanliu Fan et al. [56] introduce a monocular dense mapping system for visual–inertial odometry, optimizing IMU preintegration and applying a nonlinear optimization-based approach to improve trajectory estimation (Figure 2) and 3D reconstruction under challenging conditions. By marginalizing frames within a sliding window, it manages the computational complexity and combines an IMU and visual data to enhance the depth estimation and map reconstruction accuracy. The authors claimed the method outperforms vision-only approaches, particularly in environments with dynamic objects or weak textures, and demonstrates superior performance in comparison to existing odometry systems through evaluations of public datasets.

Two additional pioneering works are by Huang et al. [48], who introduced a VIO optimization-based online initialization and spatial–temporal calibration, and Zhou et al. [49], who introduced ‘Dplvo: Direct point-line monocular visual odometry’. The former focuses on an intricate calibration process that aligns and interpolates camera and IMU measurement data without geographical or temporal information. In contrast, the latter presents an innovative technique that leverages point and line features directly, without needing a feature descriptor, to achieve better accuracy and efficiency.

Collectively, these studies represent a robust and multifaceted exploration of traditional approaches in the realms of motion estimation, depth estimation, and scale recovery within visual odometry (VO). The methodologies vary widely, each bringing unique contributions to scientific discourse and providing promising avenues for ongoing research and development. Their collective focus on enhancing precision, robustness, and computational efficiency underscores the central challenges of the field and the diverse means by which these can be overcome.

## 5. Machine Learning-Based Approaches

Machine learning-based approaches to VO are redefining the field with innovative techniques that harness the power of neural networks. Generally, methods in this section can be classified into two distinct categories: full deep learning approaches that utilize neural networks almost exclusively, and semi-deep learning approaches that combine deep learning with more traditional computer vision techniques.

### 5.1. Full Deep Learning Approaches

Full deep learning approaches leverage the complexity and flexibility of neural networks to solve challenging VO tasks.

Yang et al. [57] pioneered a method called D3VO. This deep learning-based approach for VO estimates both camera motion and the 3D structure of the environment using just a single camera input. Comprising three specialized deep neural networks, D3VO handles depth prediction, pose estimation, and uncertainty estimation. D3VO’s method of uncertainty estimation involves predicting a posterior probability distribution for each pixel, which helps in adaptively weighting the residuals in the presence of challenging conditions, like non-Lambertian surfaces or moving objects. Despite its performance edge over existing VO methods in various benchmarks, D3VO faces significant challenges, such as the need for extensive labeled training data, complexities in securing accurate depth labels, and struggles with low-texture or featureless environments.

Ban et al. [51] contributed a unique perspective by integrating both the depth and optical flow in a deep learning-based method for VO (Figure 3). This intricate algorithm first extracts image features, which are then processed through a neural network to estimate the depth and optical flow. The combination of these elements enables the accurate computation of motion. However, a major drawback is the substantial requirement for training data, which is pivotal for effectively training the neural network.

In a novel approach, Kim et al. [53] designed a method to perform simultaneous VO, object detection, and instance segmentation. By employing a deep neural network, the method not only estimates the camera pose but also detects objects within the scene, all in real time. While promising, this approach also faces its own set of challenges, particularly the extensive need for training data and potential difficulties with occlusions and clutter.

**Figure 4:**
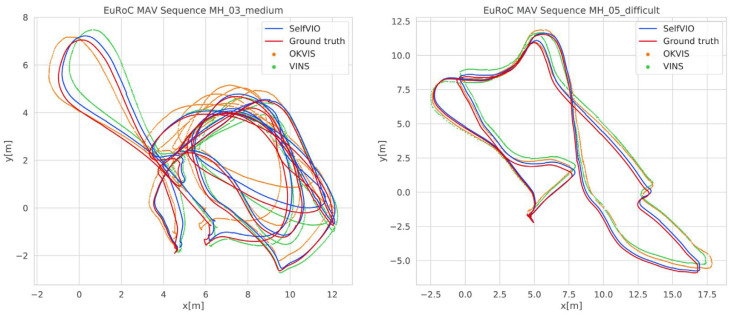
Sample trajectories comparing the unsupervised learning approach SelfVIO with monocular OKVIS, VINS, and the ground truth in meter scale using EuRoC dataset MH-03 and MH-05 sequences in [54].

A notable trend in this category involves self-supervised learning as a solution to the data scarcity problem. Many supervised methods for VIO and depth map estimation necessitate large labeled datasets. To mitigate this issue, the authors in [54] proposed a self-supervised method that leverages scene consistency in shape and lighting. Utilizing a deep neural network, this method estimates parameters such as camera pose, velocity, and depth without labeled data (Figure 4). Still, challenges persist, such as the accuracy of inertial measurements affected by noise and the depth estimation accuracy hampered by occlusions and reflective surfaces.

### 5.2. Semi-Deep Learning Approaches

Semi-deep learning approaches blend the power of deep learning with traditional techniques, leading to methods that are sometimes more adaptable to real-world constraints.

Zhou et al. [38] addressed the unique challenge of absolute scale estimation in VO using ground plane-based features. By identifying the ground plane and extracting its features, they calculated the distance to the camera, assuming certain constants such as flat ground and the known camera height. Using a convolutional neural network (CNN), the method estimates the scale factor, offering potential applications in autonomous driving and robotics.

Lin et al. [52] provided an unsupervised method for VO that ingeniously decouples camera pose estimation into separate rotation and translation components. After the initial feature extraction and essential matrix calculation, a deep learning-based network handles the distinct estimation of rotation and translation. While groundbreaking, this approach is not immune to challenges, including motion blur and changes in the lighting conditions.

Adding to the repertoire of semi-deep learning approaches, Ref. [9] introduced the Windowed Pose Optimization Network (WPO-Net) for VO estimation. In this method, features are extracted from input images, followed by relative pose computation, with a WPO-Net optimizing the pose over a sliding window. Though promising, the computational complexity of the WPO-Net stands as a substantial hurdle, potentially impeding real-time applications.

In summary, machine learning-based approaches are forging new pathways in VO, where full deep learning methods are stretching the capacities of neural networks, and semi-deep learning methods are merging traditional techniques with contemporary progressions. A salient distinction emerges in the realm of the uncertainty assessment: traditional approaches often allow for an analytical derivation of uncertainty, providing clear metrics for measurement confidence. In contrast, deep learning methods grapple with this as an open problem, with the quantification of uncertainty remaining an elusive goal in neural network-based predictions. The pursuit of uncertainty estimation in deep learning remains a vital research area, as it is critical for the reliability and safety of VO systems in practical applications. The ongoing refinement of these methods underscores a vibrant field ripe with opportunities for innovation, notwithstanding the substantial hurdles that persist.

## 6. Uncertainty of Positioning Provided by Monocular Visual Odometry

In Section 3, the uncertainty in monocular VO and its various sources were discussed. Aksoy and Alatan [58] addressed this by proposing an inertially aided visual odometry system that operates without the need for heuristics or parameter tuning. This system, leveraging inertial measurements for motion prediction and the EPnP algorithm for pose computation, minimizes assumptions and computes uncertainties for all estimated variables. They demonstrated high performance in their system, without relying on data-dependent tuning. Building on the theme of measurement precision, Ross et al. [59] delved into the intricacies of covariance estimation in a feature-based stereo visual odometry algorithm. Their approach involved learning odometry errors through Gaussian process regression (GPR), which facilitated the assessment of positioning errors alongside the monitoring of VO confidence metrics, offering insights into the uncertainty of VO position estimates. Gakne and O’Keefe [60] tackled the scale factor issue in a monocular VO using a 3D city model. They proposed a method dealing with the camera height variation to improve the accuracy of the scale factor estimation. They found that their method provided an accurate solution but up to a scale only. Choi et al. [61] proposed a robust monocular VO method for road vehicles using uncertain perspective projection. They modeled the uncertainty associated with the inverse perspective projection of image features and used a parameter space voting scheme to find a consensus on the vehicle state among tracked features. They found that their method was suitable for any standard camera that views part of the road surface in front of or behind the vehicle.

While the methods proposed in these studies differ, they all aim to improve the accuracy of monocular VO by addressing the issue of scale uncertainty. The results of these studies show that it is possible to estimate the uncertainty of positioning provided by monocular VO and improve its accuracy. However, more research is needed to develop robust and reliable methods that can be used in different applications.

The uncertainty model for monocular VO can be mathematically formulated as follows. Let $X_{t}$ represent the estimated pose of the vehicle at time *t* and $Z_{t}$ denote the visual measurements obtained from the monocular camera. The uncertainty associated with the visual measurements can be represented by the covariance matrix $R_{t}$. Additionally, the uncertainty on the vehicle motion can be captured by the covariance matrix $Q_{t}$. The relative vehicle motion can be estimated by considering the uncertainty on the backprojection of the ground plane features and the uncertainty on the vehicle motion, as proposed by Van Hamme et al. [62]. This can be mathematically expressed as:

$$X_{t}=f\left(X_{t-1},Z_{t},R_{t},Q_{t}\right)$$

where *f* represents the function that estimates the pose of the vehicle at time *t* based on the previous pose, visual measurements, and associated uncertainties. The uncertainty model integrates the uncertainty of visual measurements and the uncertainty of vehicle motion to provide a more accurate assessment of the positioning in monocular VO. The uncertainty on the backprojection of ground plane features and the uncertainty on the vehicle motion are crucial factors in accurately estimating the relative vehicle motion. The Hough-like parameter space vote is employed to extract motion parameters from the uncertainty models, contributing to the robustness and reliability of the proposed method in [61]. Despite the advancements and insights provided by the existing research, a notable gap in the literature is the lack of a comprehensive sensitivity analysis regarding the various sources of uncertainty in monocular VO. The current models and studies often overlook the full spectrum of factors that contribute to uncertainty, ranging from atmospheric conditions to sensor noise. This limitation highlights the need for a more holistic approach to uncertainty modeling in monocular VO. A complete model would not only account for the direct uncertainties in visual measurements and vehicle motion but also extend to encompass external factors, like atmospheric disturbances, lighting variations, and intrinsic sensor inaccuracies. Such a model would enable a deeper understanding of how these diverse factors interact and influence the overall uncertainty in VO systems, paving the way for the development of more sophisticated and resilient techniques that can adapt to a wider range of environmental conditions and application scenarios

## 7. Discussion

The implementation and performance of various machine learning-based methods for VO have led to interesting observations and challenges, particularly concerning feature extraction, noise sensitivity, depth estimation, and data synchronization.

The difficulty in feature extraction at high speeds is highlighted in several works [3,48,51]. This challenge is exacerbated by factors such as the optical flow on the road and increased motion blur when the vehicle moves fast. Such conditions make feature tracking an arduous task, allowing for only a limited number of valid depth estimates. Some methods have attempted to stabilize results by tuning the feature matcher for specific scenarios, like highways. Still, this often leads to complications in urban settings, where feature matches might become erratic.

Standstill detection, an essential aspect of VO, is another area fraught with difficulty. When the vehicle speed is low, errors can occur if the standstill detection is not well calibrated. The nature of the driving environment, such as open spaces where only the road is considered suitable for depth estimation, adds further complexity to the problem.

The reliance on homography decomposition, as seen in [38], has been found to be highly sensitive to noise. This sensitivity arises from the noisy feature matches obtained from low-textured road surfaces and the multitude of parameters derived from the homography matrix. The task of recovering both camera movement and ground plane geometry is a significant challenge that can affect numerical stability. Moreover, any method relying on the ground plane assumption is vulnerable to failure if the ground plane is obscured or deviates from the assumed model. This reveals the intrinsic limitation of such methods in varying environmental conditions.

A remarkable development in this field is ORB-SLAM3 [47], which has established itself as a versatile system capable of visual–inertial and multimap SLAM using various camera models. Unlike conventional VO systems, ORB-SLAM3’s ability to utilize all previous information from widely separated or prior mapping sessions has enhanced accuracy, showcasing a significant advancement in the field.

Deep learning-based approaches to VO, such as those using CNNs and RNNs, have treated VO and depth recovery predominantly as supervised learning problems [3,50,52]. While these methods excel in camera motion estimation and optical flow calculations, they are constrained by the challenge of obtaining ground truth data across diverse scenes. Such data are often hard to acquire or expensive, limiting the scalability of these approaches.

The issue of timestamp synchronization also emerges as a critical concern, as highlighted in [9]. Delays in timestamping due to factors like data transfer, sensor latency, and Operating System overhead can lead to discrepancies in visual–inertial measurements. Even with hardware time synchronization, issues like clock skew can cause mismatches between camera and IMU timestamps [63]. Moreover, synchronization challenges extend to systems using LiDAR scanners, where the alignment with corresponding camera images must be precise. Any deviation in this synchronization can lead to erroneous depth data and subsequent prediction artifacts.

In summary, the machine learning-based approaches to VO chart an intriguing course of breakthroughs and obstacles. Notable progress in employing deep learning and the advent of sophisticated systems such as ORB-SLAM3 mark the current era. Nevertheless, the domain wrestles with intricate issues concerning feature extraction, noise sensitivity, data synchronization, and the procurement of reliable ground truth data. Central to these challenges is the assessment of uncertainty: traditional VO methods could offer probabilistic insights into measurement accuracy, but the integration of uncertainty quantification within deep learning remains a nascent and critical area of research. In traditional approaches, the provided uncertainty models primarily consider sensor noise, neglecting other significant sources of uncertainty. These overlooked elements include factors such as lighting conditions and environmental parameters, which also play a crucial role in the overall accuracy and reliability of the system. A more profound understanding and effective management of uncertainty could significantly enhance the reliability and applicability of VO technologies, highlighting an essential frontier for ongoing investigative efforts. As such, there is a pressing impetus for continuous research and development to refine the robustness of VO systems and their adaptability to the unpredictable dynamics of real-world environments.

Future research in the field of VO and machine learning is set to tackle key challenges, such as improving feature extraction under difficult conditions, enhancing noise and uncertainty management, developing versatile depth estimation methods, and achieving precise data synchronization. There is a notable demand for novel feature extraction algorithms that perform well in varied environments, alongside more sophisticated models for noise filtering and uncertainty handling. Addressing depth estimation limitations and refining synchronization techniques for integrating multiple sensor inputs are also critical. Importantly, incorporating uncertainty quantification directly into deep learning models for VO could significantly boost system reliability and utility across different applications. These research directions promise to elevate the efficacy and adaptability of VO systems, making them more suited for the complexities of real-world deployment.

## 8. Conclusions

In conclusion, this paper has provided an overview of traditional techniques and deep learning-based methodologies for monocular VO, with an emphasis on displacement measurement applications. It has detailed the fundamental concepts and general procedures for VO implementation and highlighted the research challenges inherent in VO, including scale estimation and ground plane considerations. This paper has shed light on a range of methodologies, underscoring the diversity of approaches aimed at overcoming these challenges. A focus has been placed on the assessment of uncertainty in VO, acknowledging the need for further research to develop robust and reliable methods that can be used in different applications. This paper concludes by emphasizing the importance of continued research and innovation in this field, particularly in the realm of uncertainty assessment, to enhance the reliability and applicability of VO technologies. Such advancements have the potential to contribute significantly to a wide range of applications in robotics, augmented reality, and navigation systems, paving the way for more nuanced and powerful applications of VO across various domains.

## Figures and Tables

**Table 1:** A summary of the mentioned odometry techniques.

Reference	Sensor Type	Method	Environmental Structure	Open Source	Key Points
[14]	LiDAR	Bundle Adjustment	Outdoor	Yes	Using LiDAR for camera feature tracks and keyframe-based motion estimation. Labeling is used for outlier rejection and landmark weighting.
[38]	Monocular	Ground Plane-Based Deep Learning	Outdoor	No	A ground plane and camera height-based divide-and-conquer method. A scale correction strategy reduces scale drift in VO.
[42]	LiDAR	Feature Extraction	Outdoor	No	A VO algorithm using a standard front end with camera tracking relative to triangulated landmarks optimizing the camera poses and landmark map with range sensor depth information resolves monocular scale ambiguity and drift.
[43]	Monocular	Feature Extraction	Indoor	No	A VO system utilizing a downward-facing camera, feature extraction, velocity-aware masking, and nonconvex optimization, enhanced with LED illumination and a ToF sensor, for improved accuracy and efficiency in mobile robot navigation.
[44]	LiDAR	Feature Extraction	Outdoor	Yes	LVI-SAM achieves real-time state estimation and map building with high accuracy and robustness.
[45]	LiDAR	Feature Extraction	Outdoor–Indoor	No	A multi-sensor odometry system for mobile platforms that integrates visual, LiDAR, and inertial data. Real time with fixed lag smoothing.
[46]	LiDAR	Feature Extraction	Outdoor	No	A method combining LiDAR depth with monocular visual odometry, using photometric error minimization and point-line feature refinement, alongside LiDAR-based segmentation for improved pose estimation and drift reduction.
[47]	Monocular	Feature Extraction	Outdoor	Yes	The main innovation is a visual–inertial SLAM system that uses MAP estimation even during IMU initialization.
[48]	Monocular	Feature Extraction	Outdoor	No	The authors developed a lightweight scale recovery framework using an accurate ground plane estimate. The framework includes ground point extraction and aggregation algorithms for selecting high-quality ground points.
[49]	Monocular	Feature Extraction	Indoor	No	This paper presents VO using points and lines. Direct methods choose pixels with enough gradients to minimize photometric errors.
[50]	Monocular	Deep Learning Based	Outdoor	No	The approach of this paper combines unsupervised deep learning and scale recovery, which is trained with stereo image pairs but tested with monocular images.
[3]	Monocular	Deep Learning Based	Outdoor–Indoor	No	The authors proposed a self-supervised monocular depth estimation network for stereo videos, which aligns training image pairs with predictive brightness transformation parameters.
[51]	Monocular	Deep Learning Based	Outdoor	No	A VO system called DL Hybrid is proposed, which uses DL networks in image processing and geometric localization theory based on hybrid pose estimation methods.
[52]	Monocular	Deep Learning Based	Outdoor	No	The authors created a decoupled cascade structure and residual-based posture refinement in an unsupervised VO framework that estimates 3D camera positions by decoupling the rotation, translation, and scale.
[9]	Monocular	Deep Learning Based	Outdoor	No	The suggested network in this work is built on supervised learning-based approaches with a feature encoder and pose regressor that takes multiple successive two grayscale picture stacks for training and enforces composite pose restrictions.
[53]	Monocular	Deep Learning Based	Outdoor	Yes	A neural architecture that performs VO, object detection, and instance segmentation in a single thread (SimVODIS).
[54]	Monocular	Deep Learning Based	Outdoor	Yes	The proposed method is called SelfVIO, which is a self-supervised deep learning-based VO and depth map recovery method using adversarial training and self-adaptive visual sensor fusion.

## Data Availability

Data are contained within the article.
